# Mr. William J. Dornan

**Published:** 1905-04

**Authors:** 


Mr. William J. Dornan, of Philadelphia, a medical publisher of distinction, died at his home February 18, 1905, aged 75 years.

Mr. Dornan numbered many friends in the medical profession with whom he came in contact as a printer of medical works and medical society transactions.

